# Newborn pig trachea cell line cultured in air-liquid interface conditions allows a partial in vitro representation of the porcine upper airway tissue

**DOI:** 10.1186/1471-2121-15-14

**Published:** 2014-05-06

**Authors:** Mario Delgado-Ortega, Michel Olivier, Pierre-Yves Sizaret, Gaëlle Simon, François Meurens

**Affiliations:** 1 INRA, Infectiologie et Santé Publique, Nouzilly 37380, France; 2 UMR1282 Infectiologie et Santé Publique, Université François Rabelais, Tours 37000, France; 3 Département des microscopies, plate-forme R.I.O de microscopie électronique, Université François Rabelais, Tours 37000, France; 4 Anses, Ploufragan/Plouzané Laboratory, Swine Virology Immunology Unit, Ploufragan BP 53, 22440, France; 5 European University of Brittany, Rennes 35000, France; 6 Vaccine and Infectious Disease Organization-InterVac, University of Saskatchewan, 120 Veterinary Road, Saskatoon S7N 5E3 Saskatchewan, Canada

**Keywords:** Pig, Epithelial cell, Differentiation, Air-liquid interface, Trachea

## Abstract

**Background:**

The domestic pig is an excellent animal model to study human microbial diseases due to its similarity to humans in terms of anatomy, physiology, and genetics. We assessed the suitability of an *in vitro* air-liquid interface (ALI) culture system for newborn pig trachea (NPTr) cells as a practical tool for analyzing the immune response of respiratory epithelial cells to aggressors. This cell line offers a wide microbial susceptibility spectrum to both viruses and bacteria. The purpose of our study was to evaluate and characterize diverse aspects of cell differentiation using different culture media. After the NPTr cells reached confluence, the apical medium was removed and the cells were fed by medium from the basal side.

**Results:**

We assessed the cellular layer’s capacity to polarize and differentiate in ALI conditions. Using immunofluorescence and electronic microscopy we evaluated the presence of goblet and ciliated cells, the epithelial junction organization, and the transepithelial electrical resistance. We found that the cellular layer develops a variable density of mucus producing cells and acquires a transepithelial resistance. We also identified increased development of cellular junctions over the culture period. Finally, we observed variable expression of transcripts associated to proteins such as keratin 8, mucins (MUC1, MUC2, and MUC4), occludin, and villin 1.

**Conclusions:**

The culture of NPTr cells in ALI conditions allows a partial *in vitro* representation of porcine upper airway tissue that could be used to investigate some aspects of host/respiratory pathogen interactions.

## Background

The domestic pig represents an excellent animal model to study a wide range of human microbial diseases due to its similarity to humans in terms of anatomy, genetics, and physiology [1-4]. Because of this, there is an increasing need for the development of new biomedical tools in this species. The newborn pig trachea (NPTr) cell line was established from a 2-day-old piglet obtained from a specific pathogen free herd at the *Instituto Zooprofilattico Sperimentale* in Brescia [5]. The NPTr cells are non-carcinoma and non-transformed cells offering a wide microbial susceptibility spectrum which includes not only viruses [5] but also bacteria [6]. They can be used to study host/respiratory tract pathogen interactions at the cellular level. NPTr cells can also replace Madin-Darby Canine Kidney Epithelial Cells [7] for the production of viruses such as porcine influenza viruses [5]. Recently, air-liquid interface (ALI) culture of primary tracheal epithelial cells has been implemented with success in pigs [8,9]. ALI culture conditions allow a more realistic development of epithelial cells *in vitro*[10]. For instance it was shown that the pattern of expression and polarization of Toll-like receptors (TLRs) 3, 7, and 9 in cells cultured in those conditions mirrored that of the airways *ex vivo*[11] with a surface expression of these TLRs. Furthermore, ALI culture can enable the *in vitro* reconstitution of an epithelium presenting many features of the pseudo-stratified epithelium observed in the upper respiratory tract [12]. However, the use of primary epithelial cells for the ALI technique can be challenging. Contamination with fibroblasts and micro-organisms is common, requiring additional purification steps and the use of large amounts of antibiotic and anti-mycotic drugs. This creates complications in specific conditions where the use of antibiotics is not possible. Moreover, culture of primary cells requires the sacrifice of more animals than the use of a well-established and easily available cell line. Cell lines have several advantages over primary cells including their low cost, longer life span, and lower variability between passages and experiments [13]. In addition, they are generally easier to transfect and manipulate than primary cells [13]. Epithelial cells, primary cells and cell lines are usually cultured in submerged monolayers on a conventional plastic support. One of the major disadvantages of monolayer culture is the potentially irreversible and total loss of ciliated cells [12,14-16] although there are exceptions such as hamster cells which can develop cilia and goblet cell phenotypes in submerged culture [17,18]. Many studies show that ALI culture conditions are valuable to drive a differentiated phenotype [8,9,12,13,19-22] to an extent similar to that observed in native pseudo-stratified epithelium. This could be due, partially at least, to the thin layer of apical medium minimizing the diffusion barrier and resulting in an enhanced oxygen supply to a level which better meets the requirement of airway epithelial cells. Conversely it has been shown that when the cells are maintained submerged instead of at an air-liquid interface, the differentiation of epithelial cells into ciliated cells was strongly suppressed [19]. Authors showed that the removal of some substances such as epidermal growth factor, cholera toxin, and bovine pituitary extract from the media resulted in up to 4-fold increases in the number of ciliated cells detected [19]. Thus, the selection of the culture conditions has tremendous effects on the morphology and function of epithelial cells *in vitro*[23]. For all these reasons we aimed to assess the differentiation of NPTr cells cultured under ALI conditions. The ability of NPTr cells to differentiate was evaluated by light, fluorescence, transmission, and scanning electron microscopy as well as real time quantitative polymerase chain reaction (RT-qPCR). Expression of tight junction protein zonula occludens-1 *(*ZO-1) and the development of transepithelial electrical resistance (TEER) were also assessed.

## Results

### Morphological analysis of the epithelial cell layer

**Figure 1:**
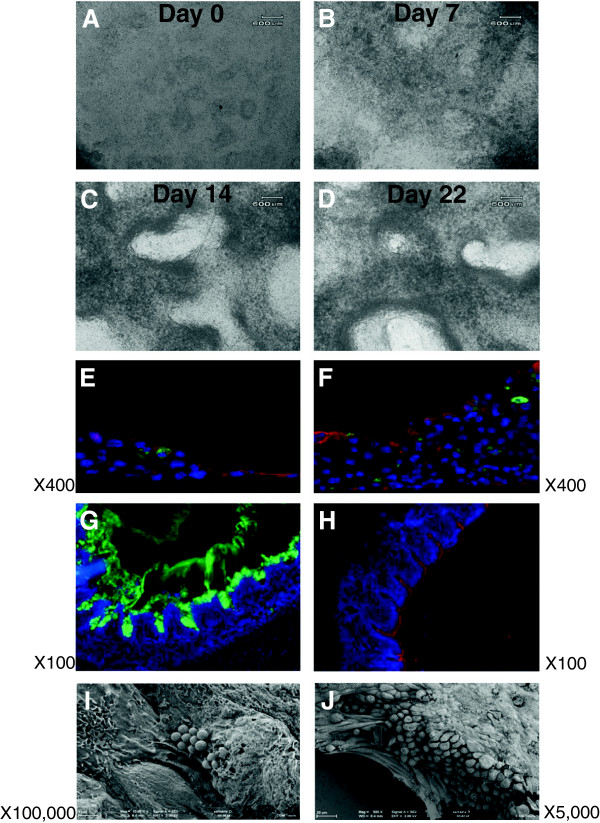
**Evolution of NPTr cells in ALI conditions over the twenty-two days of culture. A-D:** morphological evolution, representative images of two independent experiments. **E-J:** immunostaining and scanning electron microscopy assessment. NPTr cells were cultured in ALI conditions over twenty-two days and the aspect of the cellular layer was assessed by immunofluorescence and scanning electron microscopy. **E:** After reaching confluence, NPTr cells were cultured in ALI conditions for one week. **F:** After twenty-two days in ALI conditions, NPTr cells showed a thick cellular layer with the presence of numerous mucin positive cells and a delineated border of tubulin positive cells. **G** and **H:** immunostaining of the bronchial epithelium. Tissue was stained with an antibody recognizing mucin 5 AC (green, **G**) or with an antibody recognizing β-tubulin (red, **H**). **I** and **J:** Using scanning electron microscopy, a cellular layer showing a more complex topography and covered by a thick mucus layer was observed.

Cellular morphological changes were observed first by conventional light microscopy (Figure 1). Prior to confluence, NPTr cells were maintained with medium in the two chambers. After reaching full confluence, NPTr cells were cultured in ALI conditions in DMEM complemented with 10% FCS and antibiotics (Table 1) for a total of twenty-two days. At the beginning of the ALI culture NPTr cells appeared to be a homogenous population of epithelial cells with oval nuclei (Figure 1A). The confluent cells formed a monolayer of tightly packed cells. Over the subsequent days, the culture displayed darker areas, probably of stratified cells (Figure 1B), and lighter areas corresponding to less dense regions. After two and three weeks of culture, NPTr cells created multiple layers and the population seemed less homogenous with apparent increased mucus secretion (Figure 1C-D). In order to evaluate the expression of differentiation markers such as apically expressed β-tubulin (marker of ciliated epithelial cells) and mucin 5 AC (marker of goblet cells), frozen sections of NPTr cells culture were fixed and immuno-stained at the beginning (day 0) and the end (day 22) of the culture (Figure 1E-F). After seven days in ALI conditions (Figure 1E), the culture revealed a monolayer of confluent cells and the presence of multiple cell attachments suggesting the development of an increased internal complexity. After twenty-two days, the culture displayed continuous and robust cellular layers with the presence of more mucin-positive stained cells and a slightly more defined border of β-tubulin-positive cells (Figure 1F and Table 2). As a control, in respiratory pseudo-stratified epithelium collected from a two-month-old healthy pig, mucin-positive stained cells (Figure 1G) and tubulin-positive cells (Figure 1H) were easily observed. The goblet cells, mainly located in the cryptic areas of the epithelium, were capable of producing significant amounts of mucus (Figure 1G). Scanning electron microscopy confirmed the presence of some mucus particles and numerous microvilli at the apical surface at the beginning of the culture (Figure 1I). After twenty-two days of culture under ALI conditions, the surface topography was more complex showing a stratified structure covered by a mucus layer (Figure 1J). The staining for β-tubulin was globally diffused, even if some cells seemed to present a more apical staining, suggesting that villi or cilia had not developed (Figure 1F). Thus, despite the presence of microvilli, there was no evidence of cilia development at the apical surface (Figure 1J). Cell cultures using DMEM medium were monitored up to nine weeks without significant mortality of the cells. No significant differences were observed in terms of cell mortality between the second and the third week of culture where TEER was maximal.

**Table 1:** List of the different media used in the study

1)	**DMEM** + *10% FCS* + **PS**
2)	**DMEM/HAMF12** + Dexamethasone + **PS**
3)	**DMEM/HAMF12** + Epidermal growth factor + Insulin + **PS** + Selenium + Transferrin
4)	**AECM** + Bovine pituitary extract + Epidermal growth factor + Epinephrine + Hydrocortisone + Insulin + **PS** + Retinoic acid + Transferrin holo
5)	**DMEM/HAMF12** + Dexamethasone + *5% FCS* + **PS** + Retinoic acid

FCS: Fetal Calf Serum; PS: Penicillin/Streptomycin.

**Table 2:** Antibodies used for immunofluorescent staining of cultured cells

**Target**	**Antibody**	**Dilution**
**β-tubulin**	Monoclonal anti-beta-tubuline-Cy3	1/500
	clone TUB 2.1 Sigma C4585	
**Mucin**	Monoclonal anti-human gastric	1/200
	mucin 5 AC clone 45 M1 Sigma M5293	
**Z0-1**	Purified mouse anti-human ZO-1	1/100
	clone 1/ZO-1 610966 BD Biosciences	
**Mouse IgG1**	Goat anti-mouse IgG1 AF488 A21121	1/600
	Molecular Probes™ Invitrogen	
**Control**	Mouse IgG1 negative control X0931 Dako	1/50

In the experiments where conventional media was replaced by serum-free AECM media or DMEM/HAMF12 supplemented with serum, dexamethasone, and retinoic acid, the cellular layer gradually contracted, never fully covering the insert surface (data not shown), and progressively died preventing any further analyses. When serum-free supplemented DMEM/HAMF12 medium was used the cellular layer developed better (data not shown). However the culture displayed an irregular apical surface with a few mucus cells and low tubulin staining, suggesting poor cellular differentiation.

### Transepithelial electrical resistance integrity assessment of the cellular layer

**Figure 2:**
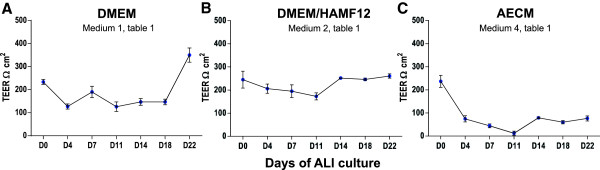
**Measurement of transepithelial electrical resistance (TEER) in NPTr cells cultured in ALI conditions.** Cells were cultured for a maximum of twenty-two days using different media **(A**, **B**, and **C)**. Data are representative of two independent experiments and are presented as means +/− SEM, (n = 6).

NPTr cells cultured with DMEM complemented with 10% FCS and antibiotics developed progressive TEER along the culture on the transwell (Figure 2A). TEER data throughout the cell culture development displayed quite stable values in the first two weeks of ALI culture (around 150 Ω cm^2^). Then, after twenty-two days TEER rose up to 350 Ω cm^2^ (Figure 2A), suggesting the formation of stronger cellular junctions. NPTr cells cultured with supplemented AECM medium failed to form a solid structure (Figure 2B). The cellular structure totally disintegrated after 14 days of ALI culture in these conditions (Figure 2B). In contrast, NPTr cells cultured with serum-free DMEM/HAMF12 medium supplemented with dexamethasone showed TEER values fluctuating around 200 Ω cm^2^ (Figure 2C) and the formation of a structure apparently more solid than the ones formed using DMEM. However, the immunostaining showed irregular apical surface with few mucus cells and low tubulin staining suggesting poor cellular differentiation (data not shown). Cells cultured with other media (Table 1) also failed to form a solid structure (data not shown).

### Zonula occludens-1 protein expression and ultrastructural analysis of newborn pig trachea cells in air-liquid interface conditions

**Figure 3:**
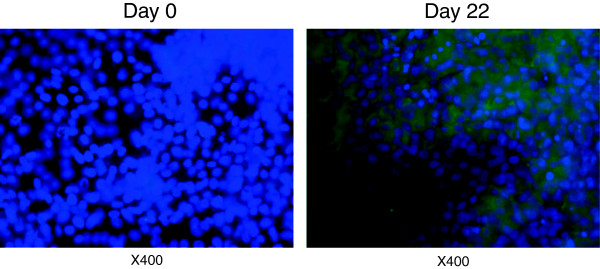
**Immunofluorescence staining of the tight junction specific protein ZO-1 in NPTr cells.** The staining was carried out at day 0 and day 22 of ALI culture.

**Figure 4:**
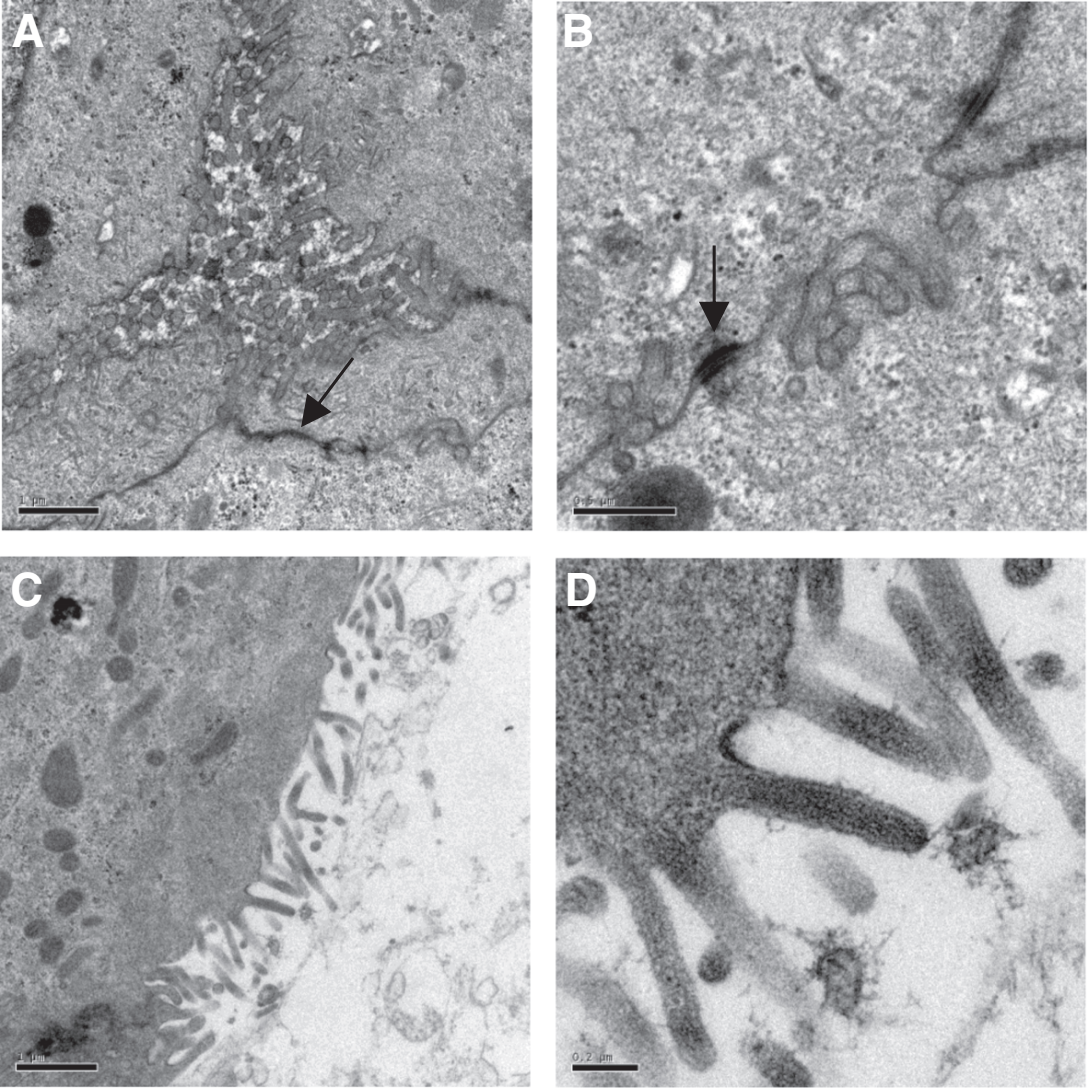
**Ultrastructural views of NPTr cells cultured in ALI conditions.** Views of the tight junction **(A**, black arrow**)** and the desmosome **(B**, black arrow**)** between adjacent cells and microvilli **(C, D)** after three weeks of culture in ALI conditions are presented. Scale bar 1 μm **(A)**, 0.5 μm **(B)**, 1 μm **(C)**, and 0.2 μm **(D)**.

Complementary immunofluorescence analysis was undertaken to evaluate the establishment of intercellular junctions by NPTr cells cultured with supplemented DMEM in ALI conditions. *Zonula occludens-1* protein (ZO-1) was identified (Figure 3). At the beginning of the culture (day 0), NPTr cells did not display any evidence of positive ZO-1 staining (Figure 3). After 14 days in ALI conditions, NPTr cultures showed positive ZO-1 spots throughout the cytoplasm of most of the cells. The staining intensified at week 3 (day 22) of culture (Figure 3). This general upward trend was correlated with the TEER findings and suggested migration of the tight junction proteins to the cell periphery. However the staining intensity was slight and ZO-1 protein did not seem to reach the cell membrane/cell-cell junctions as expected. This last observation could be linked to the use of an upright fluorescence microscope instead of a confocal microscope. Using transmission electron microscopy, well-developed cellular junctions (tight junction and desmosome) were observed after three weeks of culture under ALI conditions (Figure 4A-B). Moreover, using that technique, microvilli at the surface of the cells were identified (Figure 4C-D). However, no cilia were observed, confirming previous results.

### Expression of differentiation marker transcripts from newborn pig trachea cells cultured under two different conditions

**Figure 5:**
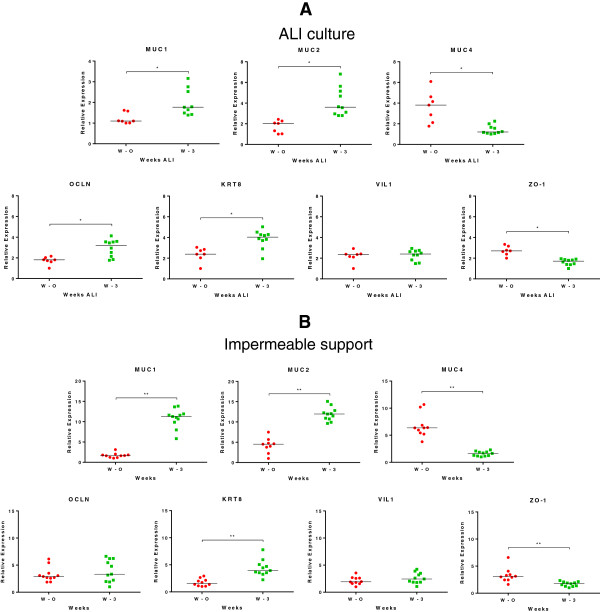
**Assessment of the mRNA expression of various cellular genes after ALI culture. (A)** Relative mRNA expressions of various cellular genes at the beginning of ALI culture (W – 0) and after three weeks of culture (W – 3). **(B)** Relative mRNA expressions of various cellular genes at the beginning of the culture on impermeable support (W – 0) and after three weeks of culture (W – 3). The median of the data is presented for a maximum of 10 independent W – 0 and W – 3 transwells. *, p < 0.05, **, p < 0.01, ns: not significant. Comparisons were carried out using non-parametric Mann-Whitney test.

To investigate the capacity of NPTr cells to differentiate in ALI conditions, the expression of differentiation and tight junction markers was analysed by RT-qPCR in cells cultured in supplemented DMEM. To normalize the mRNA relative expression, the most stable reference genes were selected among eight commonly used reference genes (Table 3). HPRT1, RPL-19, and GAPDH were the most stable genes with M values under 0.5 for cell samples (0.11, 0.11, and 0.12, respectively). In order to compare the influence of ALI conditions in cellular differentiation, a parallel experiment was performed using conventional plastic supports and again DMEM medium. Cells cultured in ALI conditions showed a significant increase in the mRNA expression of mucin 1 (MUC1), MUC2, occludin (OCLN), and keratin 8 (KRT8) (*p* < 0.05) while a significant decrease in the transcript expression of MUC4 and ZO-1 was observed (*p* < 0.05) (Figure 5A). The profile of mRNA expression in cells cultured on conventional plastic support was similar except that the expression of OCLN mRNA was not increased after 3 weeks of culture (Figure 5B). Moreover, the expression of the transcripts after three weeks of culture was even more significantly modified (*p* < 0.01) (Figure 5B).

**Table 3:** Primer sequences, annealing temperatures of primer sets (°C), expected PCR fragment sizes (bp) and accession numbers or references

**Primer name**	**Primers sequence**	**Annealing temperature (°C)**	**PCR product (bp)**	**Accession number or reference**
***ACTB***	CACGCCATCCTGCGTCTGGA AGCACCGTGTTGGCGTAGAG	63	100	[24]
***B2MI***	CAAGATAGTTAAGTGGGATCGAGAC TGGTAACATCAATACGATTTCTGA	58	161	[24]
***GAPDH***	CTTCACGACCATGGAGAAGG CCAAGCAGTTGGTGGTACAG	63	170	AF017079
***HMBS-2***	AGGATGGGCAACTCTACCTG GATGGTGGCCTGCATAGTCT	58	83	[24]
***HPRT-1***	GGACTTGAATCATGTTTGTG CAGATGTTTCCAAACTCAAC	60	91	[24]
**KRT 8**	TGACCGACGAGATCAACTTC TGATGTTCCGGTTCATCTCC	60	294	NM_001159615
**MUC1**	TAAAGAAGACGGGCTTCTGG CCGCTTTAAGCCGATCAAAC	60	134	XM_001926883
**MUC2**	ACCCGCACTACGTCACCTTC GGCAGGACACCTGGTCATTG	62	150	BX671371
**MUC4**	CTGCTCTTGGGCACTATATG CCTGTGACTGCAGAATCAAC	60	133	DQ848681
**OCLN**	CTACATAATGGGCGTCAACC GGGCTGCTCGTCATAAATAC	60	298	NM_001163647
***RPL-19***	AACTCCCGTCAGCAGATCC AGTACCCTTCCGCTTACCG	60	147	[25]
***SDHA***	CTACAAGGGGCAGGTTCTGA AAGACAACGAGGTCCAGGAG	58	141	[24]
***TBP-1***	AACAGTTCAGTAGTTATGAGCCAGA AGATGTTCTCAAACGCTTCG	60	153	[24]
**VIL1**	AGAAGTGGACGGTGCCCAAC TCTCGCCGATGAGGTAGGTG	64	273	XM_001925167
**ZO-1/TJP1**	GAGGGCATTTCCCACGTTTC GCTTTAGAGCCGAGTCCTTG	62	256	XM_003353439

Reference genes are underlined.

## Discussion

*In vitro* models using cell lines are indispensable for understanding the response of epithelium to infectious agents. In the current report we assessed the capacity of NPTr cells to polarize and differentiate when cultured under ALI conditions. Using immunofluorescence and electronic microscopy we evaluated the presence of goblet and ciliated cells, the epithelial junction organization, and the transepithelial electrical resistance. We have shown that it is possible to identify both mucin-producing cells and non-mucin-producing β-tubulin-positive cells in the NPTr population. However β-tubulin staining was quite diffuse and cilia were not observed at the apical side of the cells. Moreover, although the heterogeneity in cell population increased when the cells were cultured under ALI conditions, this condition was not absolutely necessary to generate this heterogeneity. Indeed, the two kinds of cells were detected at the beginning of the culture even when the conventional impermeable plastic support was used. NPTr cells would be, independent of the culture conditions, a heterogeneous population of cells including both cells specialized in the production of mucins, and non-mucin-producing β-tubulin-positive cells. Nevertheless, we cannot exclude the possibility of just one kind of cell only producing mucus under specific stimuli. The presence of two cell types would be interesting in the context of the study of host/pathogen interactions as viruses or bacteria sometimes discriminate between goblet and ciliated cells [20-22,25,26]. We did not culture primary trachea epithelial cells in parallel to our cultures of NPTr cells. Recently (unpublished data), some experiments involving primary bronchial epithelial cells have been initiated in the lab. Preliminary results showed significant differences between primary and NPTr cells in terms of TEER and immunostaining, strongly suggesting that the protocol and conditions used in our study could only partially account for the limited differentiation of NPTr cells.

Regarding TEER, it was observed that DMEM and serum free DMEM/HAMF12 supplemented with dexamethasone media were the only ones enabling the development of a higher resistance, with values close to the 300 Ω cm^2^ after three weeks of culture under ALI conditions. The development of high TEER values coupled with the observations we made with transmission electron microscopy and staining of ZO-1 demonstrated the development of strong intercellular junctions. The presence of multiple layers of cells also likely contributed to the increase in TEER. Curiously the mRNA expression of ZO-1 was significantly lower after three weeks of ALI culture than at the beginning, which is the opposite of what was expected. A similar result was observed also when the cells were cultured on the impermeable support. Discrepancies between the expression of ZO-1 mRNA and protein, and the TEER have also been observed by others [22]. One explanation for this could be variations in mRNA stability or protein synthesis and turn-over. The difference in the expression of OCLN mRNA observed between NPTr cells cultured on impermeable support and cells cultured under ALI conditions is probably due to a higher development of intercellular junctions when the cells were cultured on the transwells under ALI conditions. Villin -a protein associated with the actin core bundle of the brush border- transcripts (VIL1) were not expressed more after three weeks of culture on either the impermeable support or the transwell. Nossol and collaborators demonstrated variability between different cell lines, using intestinal porcine epithelial cells 1 (IPEC-1) and IPEC-J2 (isolated from the jejunum). With IPEC-1 cell culture they detected a significant increase of villin mRNA levels in conventional membrane and ALI cultures compared to impermeable dish cultures [21]. However with IPEC-J2 villin mRNA was significantly increased in cells cultured conventionally on membranes but it was not increased in cells cultured under ALI conditions, in comparison to dish culture [21]. Regarding mucins, we assessed the mRNA expression of one secreted gel-forming mucin (MUC2) and two cell surface mucins (MUC1 and MUC4) [27]. These three mucins are produced in the respiratory tract as well as in other systems [27]. The mRNA expression of both MUC1 and MUC2 was significantly increased after three weeks of culture on impermeable support and under ALI conditions while we observed a decrease in the mRNA expression of MUC4. The significant increase of MUC1 and MUC2 mRNA expression was consistent with the higher percentage of mucus-producing cells under ALI conditions after three weeks of culture. The decrease of MUC4 mRNA expression is more difficult to explain and could also be related to mRNA stability or protein synthesis and turn-over.

The cells cultured under ALI conditions with AECM and DMEM/HAMF12 without serum did not growth well nor differentiate as well as the cells cultured with DMEM supplemented with serum. Together these results show the importance of fetal calf serum in obtaining the most favorable development of NPTr cells in our ALI conditions. After three attempts using AECM medium with similar results, this medium seems more adapted for the culture of primary epithelial cells than for NPTr cell line. In previous studies focusing on the culture of respiratory tissue explants or primary respiratory epithelial cells in various species the absence of serum and retinoic acid did not prevent the harmonious development of the ciliated cells [19,20,22,28-31]. However, in two of these studies, bovine serum albumin was added to the medium [19,29]. In other studies [12,21,22], serum at various concentrations was added to the cell line or the primary cells. In our study, we were not able to fully differentiate NPTr cells under the conditions we selected. With ALI conditions using DMEM medium supplemented with serum the NPTr cells did develop intercellular junctions and cellular polarity, however “real” goblet cells and cilia did not develop. This lack of full differentiation of NPTr cells could be due to several possible factors: 1) the need to supplement the culture medium with serum, retinoic acid, or other additives despite other studies demonstrating mucociliary differentiation without this supplementation [32-34]; 2) the potential irreversible loss of the ability to develop cilia; 3) the timing we selected for our different attempts; and 4) the potential need to supplement the culture medium with still undetermined factors that would allow a full differentiation of the cells. Regarding the effect of retinoic acid, our attempts using DMEM/HAMF12 supplemented with serum, dexamethasone and retinoic acid were not convincing, as they resulted in a degraded cellular monolayer. The origin of the serum could also be particularly critical as recently demonstrated with porcine cell line IPEC-J2 [35]. Authors showed that porcine serum was allowing a better differentiation of the cell line than previously used bovine serum [35].

## Conclusions

Briefly our data showed that both mucus-producing cells and non-mucus-producing β-tubulin-positive epithelial cells were already detectable at the beginning of the ALI culture with an increase in the number of mucus-producing cells after a few weeks under ALI conditions. Transepithelial electrical resistance increased slowly over time and strong intercellular junctions were observed at the end of the culture period. Nevertheless, even when well-developed microvilli were identified on the cells, no cilia were detected. Moreover, the generated epithelium was globally more similar to a stratified squamous than a pseudo-stratified epithelium. In our study, the culture of NPTr cells in ALI conditions enabled the development of a system intermediate between the conventional cell line culture and the culture of primary tracheal epithelial cells in ALI conditions. However, it was not possible to mimic the pseudo-stratified epithelium seen with primary epithelial cells. Improvement of the cell culture conditions may allow the full differentiation of NPTr cells to both ciliated and goblet cells even if we cannot exclude the possibility that NPTr cells somehow have lost definitely the capacity to develop cilia.

## Methods

### Culture support

Culture support was prepared according to Bals and collaborators [36] except that 50 μl of 0.01% collagen solution (Sigma–Aldrich, Saint-Quentin, France) were used to coat a 6.54 mm ThinCert™ - TC Inserts (Greiner bio-one, Courtaboeuf, France).

### Newborn pig trachea cell culture

The NPTr cells [5] (between 30 and 50 passages) were cultured in Dulbecco’s modified Eagle medium (DMEM) (Invitrogen, Cergy Pontoise, France) supplemented with 10% fetal calf serum (FCS) (Sigma-Aldrich), 20 IU/ml of penicillin and 20 mg/ml of streptomycin (Invitrogen). Cells were plated onto 24-well plastic plates (Greiner bio-one, Courtaboeuf, France) and incubated at 37°C in 5% CO_2_ in a humidified atmosphere. Sub-passages were made when cells reached 100% confluence. After trypsinization, collected cells were seeded onto coated ThinCert™ - TC Inserts (Greiner bio-one). A total of 0.8 ml of fresh medium was added to the lower reservoir and 0.25 ml of a 10^5^ cells/ml suspension was added to the upper reservoir. As a control, cells were also plated onto conventional 24-well plastic plates for twenty-two days.

### Culture after seeding cells on the insert

After seven days of culture at 37°C in 5% CO_2_ in a humidified atmosphere, when cells were completely confluent, medium was removed from the upper reservoir. The cells were gently washed with Ca/Mg-free phosphate buffered saline (PBS) every two days at the apical side. Half of the basolateral culture medium was replaced every other day. The culture was kept in ALI conditions for twenty-two days.

Parallel experiments were carried out in order to evaluate the cells’ capacity to fully differentiate using other culture media. NPTr cells were cultured with different types of media (Table 1). Again, after seven days of culture, when cells were completely confluent, the apical medium was removed. The culture medium in the lower reservoir was replaced by serum-free 50% Dulbecco's Modified Eagle's Medium (DMEM)–50% DMEM/Ham's F-12 (HAMF12) medium (Sigma-Aldrich) supplemented with 10^−7^ M dexamethasone (Sigma-Aldrich), 20 IU/ml of penicillin and 20 mg/ml of streptomycin (Invitrogen) or DMEM/HAMF12 supplemented with insulin (5 mg/ml), transferrin (5 mg/ml), selenium (5 ng/ml), epidermal growth factor (5 mg/ml) (all supplied by Sigma-Aldrich), and 20 IU/ml of penicillin and 20 mg/ml of streptomycin (Table 1). Every two days, the basal medium was changed and the apical surface washed with Ca/Mg-free PBS. The cultures were kept for twenty-two days in ALI conditions to induce cell differentiation. Finally, another experiment was performed to evaluate the impact of retinoic acid on NPTr cell differentiation. The procedure was identical to the one described above except that the culture medium in the lower reservoir was replaced either by serum free airway epithelial cell medium (AECM) (Promocell, Heidelberg, Germany) supplemented as recommended by the supplier with bovine pituitary extract (0.004 ml/ml), epidermal growth factor (10 ng/ml), insulin (5 μg/ml), hydrocortisone (0.5 μg/ml), epinephrine (0.5 μg/ml), triiodo-L-thyronine (6.7 ng/ml), transferrin holo (human) (10 μg/ml), and retinoic acid (0.1 ng/ml) (all supplied by Promocell) or DMEM/HAMF12 supplemented with 5% FCS, 20 IU/ml of penicillin and 20 mg/ml of streptomycin, dexamethasone 10^−7^ M, and retinoic acid (0.1 ng/ml) (Table 1).

### Transepithelial electrical resistance measurements

Transepithelial electrical resistance (TEER) measurement provides an indirect measure of the formation of tight junctions [37]. Among the cell-cell junctions (tight junctions, adherens junctions, gap junctions, and desmosomes), tight junctions are the most important for maintaining epithelial integrity. TEER is also used as a marker of disruption of epithelial cells. TEER was measured using a MILLICELL® ERS volt-ohm meter (Millipore, Molsheim, France). On day 0 and every fourth day up to day 22 in ALI conditions, 150 μl of medium was added apically into the insert and the measurement taken. Apical medium was then aspirated to restore ALI conditions. Prior to testing the culture’s TEER an empty culture insert was used as a blank and subtracted from each subsequent sample reading. Data are presented as resistance values (Ω cm^2^).

### Immunofluorescence staining

Immunofluorescence staining was performed directly on cells cultured onto the ThinCert™ - TC Inserts (Greiner bio-one), on ThinCert™ - TC Insert frozen sections and on lung tissue frozen sections as described below.

#### Inserts

Cell cultures were washed three times with PBS prior to fixation for 15 min with 3% paraformaldehyde (Sigma-Aldrich). After one wash with PBS containing 0.1 M glycin (Fisher Scientific, Illkirch, France) cells were treated for permeabilization with 0.2% Triton X-100 (Sigma-Aldrich) over 15 min. Finally, inserts were washed three times with Ca/Mg-free PBS before staining.

#### Insert frozen sections

Insert membranes were removed from the ThinCert™ membrane supports, then immersed in Tissue-Tek® O.C.T. Compound (Sakura Finetek, Flemingweg, The Netherlands), snap-frozen, and stored at −80°C. Serial transverse sections (7 μm thick) of the membrane were cut at −20°C using a LEICA CM3050 microtome (Leica, Nanterre, France),collected onto treated glass slides (SuperFrost Plus, Menzel-Glaser, Braunschweig, Germany), air-dried, fixed in acetone (Sigma-Aldrich) for 10 min at 4°C, and then stored at −80°C until use. Insert frozen sections were washed three times with Ca/Mg-free PBS before staining.

#### Lung tissue frozen sections

Small pieces of lung tissue (6 mm × 6 mm) were collected from a two-month-old healthy pig provided by INRA experimental unit (Nouzilly, France). The pig was cared for in accordance with the guidelines of the Institutional Animal Care and Use committee at INRA. The pieces were then immersed in Tissue-Tek® O.C.T. Compound (Sakura Finetek), snap-frozen, and stored at −80°C. Serial transverse sections (7 μm thick) of the membrane were cut at −20°C using a LEICA CM3050 microtome and treated as described above for the insert frozen sections.

#### Staining

In the case of filter cultures, the reagents were added to the apical filter chamber. Each incubation period with the selected antibodies was performed at room temperature for 20 min in the dark. The goblet cells were stained indirectly by using monoclonal anti-human gastric mucin 5 AC clone 45 M1 antibodies (dilution 1/200) (Sigma-Aldrich) followed by AF488-labeled secondary antibodies (dilution 1/600) (Invitrogen) (see Table 2). Tight junctions were stained with purified monoclonal mouse anti-human ZO-1 antibodies (dilution 1/100) (BD Biosciences, Rungis, France). For cilium staining, cells were treated with Cy3-labeled monoclonal antibodies recognizing β-tubulin (dilution 1/500) (clone TUB 2.1, Sigma-Aldrich). β-tubulin is often expressed as a cytoskeletal protein, however, its apical expression is a marker of ciliated cells [38]. 4’, 6’-diamidino-2-phenylindole (DAPI) (Life Technologies Inc., Carlsbad, CA, USA) at 0.5 μg/ml was used as counterstaining before the cells were washed three times with Ca/Mg-free PBS. Controls were incubated with primary isotype control antibodies followed by secondary antibodies (Table 2). All samples were observed with a Nikon Eclipse 80i microscope connected to Nikon intensilight C-HGF and the imaging software NIS Elements D (Nikon Instruments Europe BV, Amsterdam, The Netherlands).

### Transmission electron microscopy

The filter membranes with NPTr cells were fixed by incubation for 24 h in 4% paraformaldehyde and 1% glutaraldehyde in 0.1 M phosphate buffer (pH 7.4) (Sigma-Aldrich) and post-fixed by incubation for 1 h with 2% osmium tetroxide (Electron Microscopy Science, Hatfield, PA, USA). They were then dehydrated in a graded series of ethanol solutions, cleared in propylene oxide, and embedded in Epon resin (Sigma-Aldrich) which was allowed to polymerize for 48 h at 60°C. Ultra-thin sections were cut and placed on 300 mesh copper grids and then stained with 5% uranyl acetate and 5% lead citrate (Sigma-Aldrich). The grids were then observed with Jeol 1230 TEM (Tokyo, Japan) connected to a Gatan slow scan digital camera and digital micrograph software (Gatan, Pleasanton, CA, US) for image acquisition.

### Scanning electron microscopy

The filter membranes with NPTr cells were washed in PBS, fixed in 4% paraformaldehyde (Sigma-Aldrich) and 1% glutaraldehyde in 0.1 M phosphate buffer (pH 7.4) (Sigma-Aldrich) and post-fixed by incubation for 1 h with 2% osmium tetroxide. Then, specimens were dehydrated in a graded series of acetone and dried in hexa-methyl-disilazan solution (HMDS) (Sigma-Aldrich). Dried specimens were coated with a thin layer of platinum with ion beam coater PECS (Gatan France, Evry, France) and observed with Zeiss Ultra + Field Emission Gun Scanning electron microscope (FEGSEM) (Carl Zeiss S.A.S, Le Pecq, France).

### Real time polymerase chain reaction assays and validation of reference genes

NPTr cells were lysed and total RNA was isolated using RNeasy Mini kit (Quiagen, Courtaboeuf, France). Quantitative real-time PCR (qPCR) was performed using cDNA synthesized as previously described [39]. Primers were designed using Clone Manager 9 (Scientific & Educational Software, Cary, NC, USA) and were purchased from Eurogentec (Liège, Belgium) (Table 3). Diluted cDNA (10X) was combined with primer/probe sets and IQ SYBR Green Supermix (Bio-Rad, Hercules, CA, USA) according to the manufacturer’s recommendations. The qPCR conditions were 98°C for 30 seconds, followed by 37 cycles with denaturation at 95°C for 15 seconds and annealing/elongation for 30 seconds (annealing temperature, Table 3). Real time assays were run on a Bio-Rad Chromo 4 (Bio-Rad, Hercules, CA, USA). The specificity of the qPCR reactions was assessed by analyzing the melting curves of the products and size verification of the amplicons. To minimize sample variations, we used an identical amount of cells and high quality RNA. The quality of RNA was assessed by capillary electrophoresis (Agilent 2100 Bioanalyzer, Agilent Technologies, Massy, France) and RNA integrity numbers (RIN) were calculated. RIN were always ≥8.7 demonstrating the high quality of the RNA. Samples were normalized internally using simultaneously the average *cycle quantification* (*Cq*) of the three most suitable reference genes in each sample to avoid any artifact of variation in the target gene. These three most suitable reference genes were selected among eight commonly used reference genes which were investigated in each tissue using qPCR with SYBR green. The genes included beta-actin (*ACTB*), beta-2-microglobulin (*B2MI*), glyceraldehyde-3-phosphate dehydrogenase (*GAPDH*), hydroxymethylbilane synthase (*HMBS*), hypoxanthine phosphoribosyltransferase-1 (*HPRT-1*), ribosomal protein L-19 (*RPL-19*), succinate dehydrogenase complex subunit A (*SDHA*) and TATA box binding protein 1 (*TPB-1*). The stability of these reference genes in all the selected tissues was investigated using the geNorm application [40]. The threshold for eliminating a gene was M ≥0.5 as recommended [41]. The correlation coefficients of the standard curves were >0.995 and the concentration of the test samples was calculated from the standard curves, according to the formula y = −M*Cq + B, where M is the slope of the curve, *Cq* the first positive second derivative maximum of amplification curve calculated using PCR Miner [42] and *B* the y-axis intercept. All qPCRs displayed efficiency between 90% and 110%. Expression data are expressed as relative values after Genex macro analysis (Bio-Rad, Hercules, CA, USA) [40].

### Statistical analysis

Data for the comparison of differences in relative mRNA expression between NPTr cells (W – 0 and W – 3) were expressed as relative values. Because data were independent and non-normally distributed, the Mann–Whitney test was selected for statistical analysis (GraphPad Prism software version 3.00, GraphPad Software Inc., San Diego, CA, USA). Differences between groups were considered significant when *p* < 0.05.

## Competing interests

The authors declare that they have no competing interests.

## Authors’ contributions

MDO and MO carried out most of the experiments, participated in the analysis of the data and drafted the manuscript. PYS performed the electronic microscopy analysis. GS provided the NPTr cells, participated in the design of some experiments, and helped to draft the manuscript. FM conceived the study, actively participated in its design and coordination, analyzed the data, drafted and revised the manuscript. All authors read and approved the final manuscript.
